# Dermatomyositis and supraventricular tachycardia

**DOI:** 10.1186/1755-7682-1-25

**Published:** 2008-11-13

**Authors:** Abhijeet Dhoble, Chethan Puttarajappa, Alan Neiberg

**Affiliations:** 1 Department of Internal Medicine, Michigan State University, East Lansing, Michigan, USA

## Abstract

**Background:**

Dermatomyositis is an idiopathic inflammatory myopathy, often associated with an underlying malignancy. Its prevalence rate is approximately one per 100,000 in the general population, and is even rarer without evidence of a cancer. Dermatomyositis rarely involves myocardial muscle fibers, but has shown to be associated with cardiac arrhythmias.

**Case Presentation:**

We present a case of a young female patient with known history of dermatomyositis who presented to hospital with a flare up of her disease. She also complained of paroxysms of palpitation. Telemetry monitoring revealed several episodes of paroxysmal supraventricular tachycardia with heart rate reaching up to 220 beats per minute.

**Conclusion:**

Cardiac involvement in dermatomyositis is a very rare, but well known entity. Dermatomyositis patients with palpitations should be monitored on a Holter monitor, and appropriate therapy initiated if found to have a significant arrhythmia.

## Background

Dermatomyositis (DM) is a type of idiopathic inflammatory myopathy [1,2]. Its prevalence rate is approximately one per 100,000 in the general population with a female to male predominance of about 2:1. DM is usually associated with an underlying malignancy, and its prevalence is even rarer without coexistent cancer [1-4]. DM is characterized by immune complex deposition in the vessels and is considered to be in part a complement-mediated vasculopathy [1,5].

DM rarely involves myocardial muscle fibers, but cardiac involvement is a well described entity in this disorder. It has shown to be associated with various arrhythmias including ventricular and supraventricular tachycardia [6,7]. We present a similar case here, followed by a discussion.

## Case presentation

A 27 year old woman presented with intense pruritis for two weeks. She also complained of myalgia and generalized weakness of equal duration. Her past history was significant for rheumatoid arthritis and dermatomyositis, and was on medications for that. She had stopped her medicines four weeks ago, which included prednisone, azathioprine, and hydroxychloroquine. After further questioning, she mentioned that she gets paroxysms of palpitations occasionally associated with dizziness. She denied any syncopal episodes, chest pain, or headache along with these symptoms.

**Figure 1:**
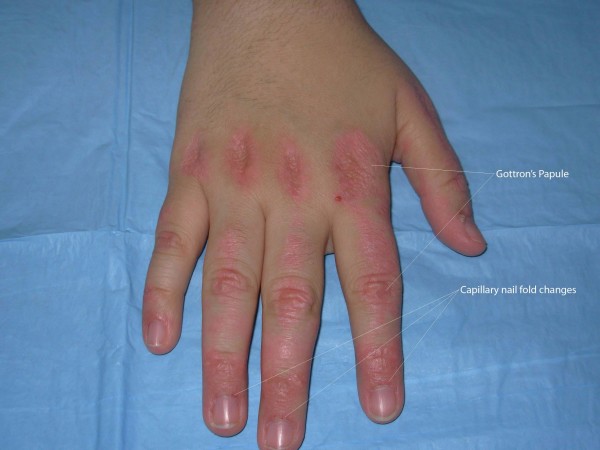
Erythematous, scaly eruption (Gottron's papules) over the metacarpophalangeal and interphalangeal joints. It also shows abnormal capillary nail bed change.

**Figure 2:**
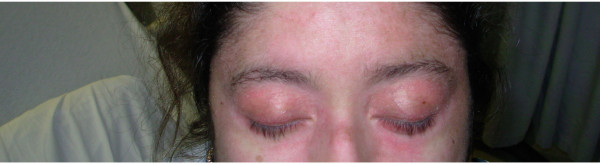
Violaceous eruption (heliotrope rash) on the upper eyelids, accompanied by eyelid swelling.

**Figure 3:**
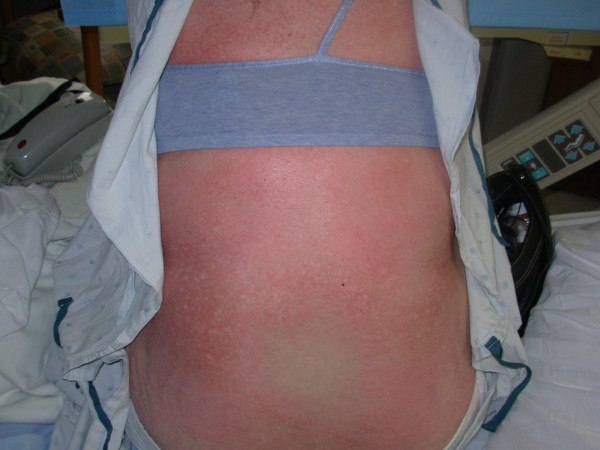
Diffuse erythroderma.

**Figure 4:**
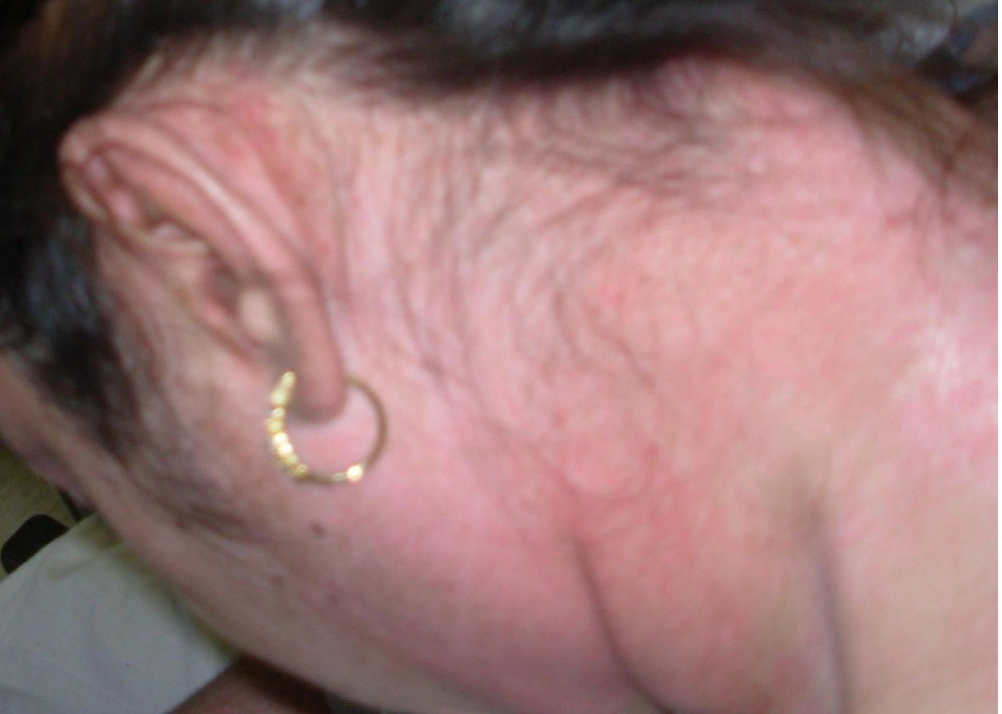
Psoriasiform changes of scalp.

Physical examination revealed erythematous, scaly eruption, also known as Gottron's papules over the metacarpophalangeal and interphalangeal joints (figure 1). She also had violaceous eruption (heliotrope rash) on the upper eyelids, accompanied by eyelid swelling (figure 2), diffuse erythroderma (figure 3), psoriasiform changes of scalp (figure 4), and abnormal capillary nail bed change (figure 1). At the time of initial examination, she had unremarkable vital signs, respiratory and cardiovascular examination. Laboratory investigations including Creatnine Kinase level, troponins, metabolic panel, and complete hemogram were within normal limits. Baseline electrocardiogram (EKG) showed normal sinus rhythm with no abnormal changes. Chest radiograph did not show cardiomegaly or infiltrates.

**Figure 5:**
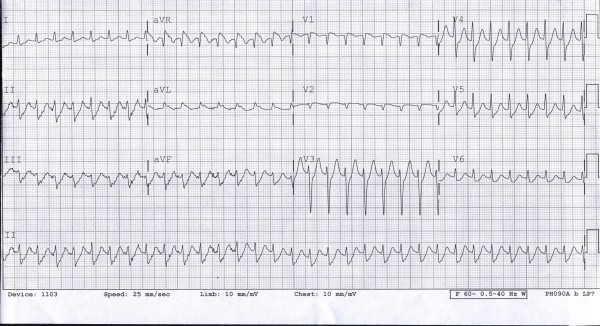
An EKG showing atrio-ventricular node re-entry tachycardia with rate of 200 beats per minute.

Telemetry monitoring revealed several episodes of paroxysmal supraventricular tachycardia with heart rate reaching up to 220 beats per minute (bpm). EKG was obtained during one such episode which showed supraventricular tachycardia with rate of 200 bpm (figure 5). A two-dimensional echocardiogram was obtained which was within normal limits. Additional imaging studies including cardiac magnetic resonance imaging and cardiac computed tomogram were not performed due to low yield. In addition to high dose oral prednisone, she was immediately started back on her other medications. She was given metoprolol for the tachyarrhythmia. Telemetry monitoring did not show any recurrent episodes of arrhythmia over the next 24 hours. Rash and other symptoms improved considerably over the next two days, and she was discharged home on the above medications.

## Discussion

DM is associated with a variety of characteristic skin manifestations, including Gottron's sign, the shawl sign, the heliotrope rash, and a generalized erythroderma. This patient manifested all classic skin signs with her flare up of DM [2,3].

Cardiac involvement in DM is a very rare, but well described entity. These patients can manifest with AV blocks and ventricular or supraventricular tachyarrhythmias (VT and SVT). Postulated mechanisms include: 1) Formation of re-entry circuits 2) Myocardial fibrosis due to recurrent inflammation, and 3) Active inflammatory myocarditis [6-12]. Very few case reports and series are mentioned in the literature regarding DM and tachyarrhythmias [6,8,10]. Few autopsy based studies have tried to establish this association of cardiac involvement in DM patients retrospectively [6,12].

Treatment should be individualized, and close follow up is necessary. DM patients with SVT usually respond well to medical management [8,11]. We think that the cause of SVT in our patient was local active inflammation. After her discharge, she was monitored on Holter monitor for a month which failed to show SVT. This supports our hypothesis that active inflammation played a major role in this patient's SVT. We gradually took her off metoprolol, and patient denied any further episodes of palpitations at six month follow up.

## Conclusion

Cardiac involvement in DM is a very rare, but well known entity [7,8]. A thorough history for cardiac symptoms is very important to prevent any future major cardiac event. DM patients with palpitations should be monitored on a Holter, and appropriate therapy initiated if found to have a significant arrhythmia.

## Consent

An informed consent was obtained from the patient for publication of this case report and accompanying images in a medical journal. A copy of the written consent is available for review by the Editor-in-Chief of this journal.

## Competing interests

The authors declare that they have no competing interests.

## Authors' contributions

AD was involved in acquisition of images and preparation of manuscript. CP was involved in collecting patient data, literature review, and revision of manuscript. AN was involved in patient care and critically revising the content of manuscript. All authors read and approved the final manuscript.
